# Mycotoxins in Conversation With Bacteria and Fungi

**DOI:** 10.3389/fmicb.2019.00403

**Published:** 2019-03-19

**Authors:** Nandhitha Venkatesh, Nancy P. Keller

**Affiliations:** ^1^Department of Plant Pathology, University of Wisconsin—Madison, Madison, WI, United States; ^2^Department of Medical Microbiology and Immunology, University of Wisconsin—Madison, Madison, WI, United States; ^3^Department of Bacteriology, University of Wisconsin—Madison, Madison, WI, United States

**Keywords:** Mycotoxins–*Fusarium*, bacterial-fungal interaction (BFI), mycotoxin ecological role, microbial interaction, microbial communication

## Abstract

An important goal of the mycotoxin research community is to develop comprehensive strategies for mycotoxin control and detoxification. Although significant progress has been made in devising such strategies, yet, there are barriers to overcome and gaps to fill in order to design effective mycotoxin management techniques. This is in part due to a lack of understanding of why fungi produce these toxic metabolites. Here we present cumulative evidence from the literature that indicates an important ecological role for mycotoxins, with particular focus on *Fusarium* mycotoxins. Further, we suggest that understanding how mycotoxin levels are regulated by microbial encounters can offer novel insights for mycotoxin control in food and feed. Microbial degradation of mycotoxins provides a wealth of chemical information that can be harnessed for large-scale mycotoxin detoxification efforts.

## Introduction

The term mycotoxin refers to harmful secondary metabolites produced by fungi in food and feed products that negatively impact animal and human health, by themselves or through synergistic interactions with each other. Initially thought to be waste products, fungal secondary metabolites are now considered as important players in ecological settings. Some metabolites provide protection from physical damage. For example, spore melanins have been demonstrated to provide protection against ionizing radiation as well as oxidizing agents in addition to acting as virulence factors (Eisenman and Casadevall, 2012). Some fungal metabolites provide protection against other microbes, helping the producing fungus to secure its environmental niche. Gliotoxin, an antifungal produced by several fungi, is a virulence factor of the human pathogen *Aspergillus fumigatus* (Scharf et al., 2016).

**Figure 1:**
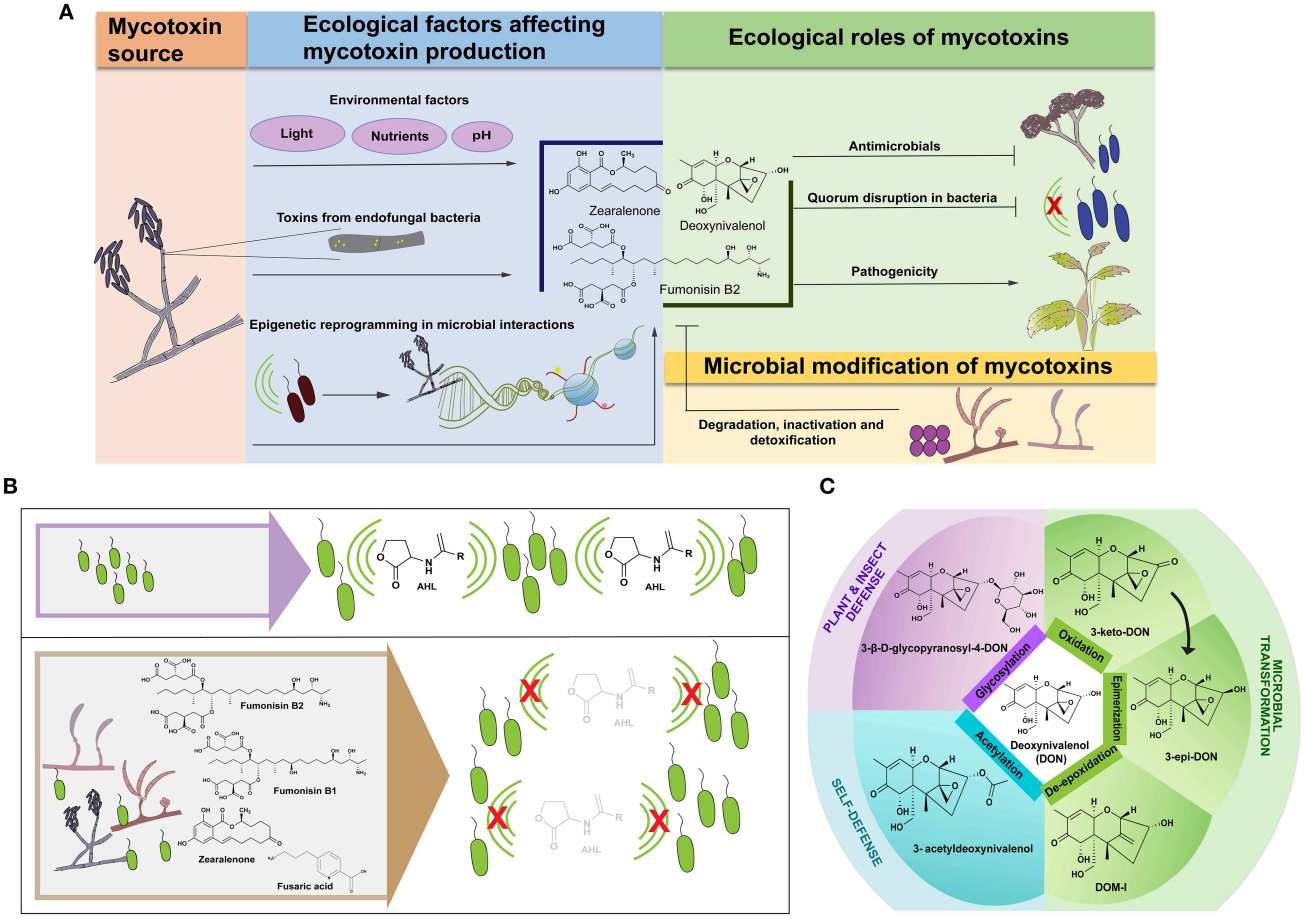
Mycotoxins in microbial interactions. **(A)** Mycotoxins in the ecological landscape—Bacterial-fungal interactions influence mycotoxin production in addition to environmental factors (blue box). In some cases, the toxin may be produced by an endofungal bacterium (shown as yellow circular bacteria residing in the gray hypha). Microbial interactions may alter epigenetic modifications (indicated by DNA wrapped around blue histones where the yellow and pink shapes represent epigenetic modifications in the histone tail) that regulate mycotoxin production. These secreted mycotoxins in turn play vital roles in shaping ecological niches (green box) by acting as antimicrobials in addition to inhibiting bacterial quorum-based communication (bacterial communication is represented with green arcs; the red “X” indicates disruption of communication). Mycotoxins also alter the pathogenic abilities of the producing fungus that in turn may influence the microbial communities in the niche. In such niches, microbes (represented by fungi and spherical bacteria in the yellow box) can detoxify, degrade, and inactivate mycotoxins. **(B)** *Fusarium* mycotoxins inhibit acyl homoserine lactone (AHL)-based quorum sensing in bacteria. (Top panel) The green arcs indicate active quorum-based communication in a bacterial population. (Bottom panel) The left end of the lower box shows *Fusarium* spp. co-occurring with bacteria. The right end shows mycotoxins produced by *Fusarium* spp. that contribute to quorum quenching in the bacterial population, indicated by the red “X” over the green arcs. **(C)** Detoxification processes of deoxynivalenol (DON) and their products that have been tested to show reduced toxicity compared to DON. The arrow from 3-keto-DON to 3-epi-DON indicates that formation of the epimer proceeds with 3-keto-DON as an intermediate.

This notion of ecological function is applicable to all fungal secondary metabolites including mycotoxins (Figure 1A). *Fusarium* species comprise a well-known group of soil-borne microbes that are infamous for their ability to make many potent mycotoxins (Table 1). In soil and host environments, *Fusarium* spp. engage in intimate associations with other microbes. This review will examine when, where and why mycotoxins are made, highlighting the ecological importance of mycotoxins with a special emphasis on the involvement of *Fusarium* mycotoxins in bacterial-fungal interactions.

**Table 1:** List of different mycotoxins and their chemical classes, the *Fusarium* species identified as producers of each mycotoxin, and corresponding reported activities.

**Mycotoxin**	**Producers identified**	**Chemical class**	**Reported activity**	**References**
HT2 toxin	*F*. *langsethiae*^1^, *F*. *sporotrichoides*^1^, *F. culmorum*^1^, *F. poae*^1, 8^, *F. sporotrichoides*^3^, *F. acuminatum*^8^, *F. chlamydosporum*^8^	Type-A trichothecene	Hematotoxicity^25^, myelototoxicty^25^	^1^ Shi et al., 2016 ^3^Thrane, 1986 ^8^Beukes et al., 2017 ^25^Lautraite et al., 1996
T2 toxin	*F. langsethiae*^1^, *F. sporotrichoides*^1^, *F. culmorum*^1, 8^, *F. poae*^1, 8^, *F. sporotrichoides*^3^, *F. acuminatum*^9^, *F. chlamydosporum*^8^	Type-A trichothecene	Hematoxicity^35^, myelototoxicity^35^	^1^ Shi et al., 2016 ^3^Thrane, 1986 ^8^Beukes et al., 2017 ^9^Bottalico and Perrone, 2002 ^35^Chilaka et al., 2017
Neosolaniol	*F. langsethiae*^1^, *F. sporotrichoides*^1, 3^, *F. culmorum*^1^, *F. poae*^1^, *F. meridionale*^1^, *F. acuminatum*^9^	Type-A trichothecene	Hematotoxicity^34^	^1^ Shi et al., 2016 ^3^ Thrane, 1986 ^9^ Bottalico and Perrone, 2002 ^34^ Janse van Rensburg et al., 1987
Diacetoxyscirpenol	*F. langsethiae*^1^, *F. sporotrichoides*^1, 5^, *F. polyphialadicum*^1^, *F. poae*^1, 5^, *F. equiseti*^4^, *F. chlamydosporum*^5^, *F. avenaceum*^5^, *F. semitectum*^5^, *F. acuminatum*^5^, *F. compactum*^5^, *F. sambucinum*^5^, *F. venenatum*^5^, *F. culmorum*^5^, *F. graminearum*^5^, *F. crookwellense*^5^	Type-A trichothecene	Hematotoxicity^34^, teratogenicity^39^	^1^ Shi et al., 2016 ^4^ Hestbjerg et al., 2002 ^5^ Schollenberger et al., 2007 ^34^ Janse van Rensburg et al., 1987 ^39^ Mayura et al., 1987
Deoxynivalenol	*F. graminearum*^1^, *F. culmorum*^9^, *F. acuminatum*^8^, *F. crookwellense*^8^, *F. pseudograminearum*^8^, *F. semitectum*^8^	Type-B trichothecene	Cytotoxicity^28^, endocrine disruption^26^, immune modulation^26^, developmental and reproductive toxicity^26^, genotoxicity^26^	^1^ Shi et al., 2016 ^8^ Beukes et al., 2017 ^9^ Bottalico and Perrone, 2002 ^26^ Knutsen et al., 2017 ^28^ Alassane-Kpembi et al., 2013
Nivalenol	*F. culmorum*^1, 9^, *F. poae*^1, 8^, *F. meridionale*^1, 8^, *F. graminearum*^8^, *F. equiseti*^4^, *F. crookwellense*^9^, *F. pseudograminearum*^8^, *F. semitectum*^8^, *F. acaciae-mearnsii*^8^, *F. brasilicum*^8^, *F. cortaderiae*^8^	Type-B trichothecene	Cytotoxicity^28^, hematotoxicity^35^, immunotoxicity^35^	^1^ Shi et al., 2016 ^4^ Hestbjerg et al., 2002 ^8^ Beukes et al., 2017 ^9^ Bottalico and Perrone, 2002 ^28^ Alassane-Kpembi et al., 2013 ^35^ Chilaka et al., 2017
Fusarenon-X	*F. culmorum*^1, 8^, *F. poae*^1, 8^, *F. meridionale*^1^, *F. graminearum*^3^, *F. equiseti*^30^, *F. crookwellense*^9^, *F. pseudograminearum*^8^	Type-B trichothecene	Genotoxicity^28^, cytotoxicity^28^	1. Shi et al., 2016 ^3^ Thrane, 1986 ^8^ Beukes et al., 2017 ^9^ Bottalico and Perrone, 2002 ^38^ Alassane-Kpembi et al., 2013 ^30^Jestoi, 2008
15-ADON	*F. graminearum*^1, 8^, *F. boothi*^8^	Type-B trichothecene	Cytotoxicity^28^	^1^ Shi et al., 2016 ^8^ Beukes et al., 2017 ^28^ Alassane-Kpembi et al., 2013
3-ADON	*F. graminearum*^1, 8^, *F. culmorum*^4^, *F. acaciae-mearnsii*^8^, *F. brasilicum*^8^, *F. cortaderiae*^8^	Type-B trichothecene	Cytotoxicity^28^	^1^ Shi et al., 2016 ^4^ Hestbjerg et al., 2002 ^8^ Beukes et al., 2017 ^28^ Alassane-Kpembi et al., 2013
Beauvericin	*F. acuminatum*^8^, *F. anthophilum*^8^, *F. avenaceum*^8^, *F. globosum*^8^, *F. fujikuroi*^8^, *F. nygamai*^8^, *F. oxysporum*^8^, *F. poae*^8^, *F. proliferatum*^8^, *F. semitectum*^8^, *F. subglutinans*^8^, *F. temperatum*^8^, *F. verticillioides*^8^, *F. acutatum*^30^, *F. beomiforme*^30^, *F. circinatum*^30^, *F. concentricum*^30^, *F. dlamini*^30^, *F. equiseti*^30^, *F. guttiforme*^30^ *F. konzum*^30^, *F. langsethiae*^30^, *F. longipes*^30^, *F. pseudoanthophilum*^30^, *F. sambucinum*^30^*, F. sporotrichioides*^30^, *F. tricinctum*^30^	Non- ribosomal peptide	Antimicrobial activity^30^, insecticidal activity^30^, cytotoxicity^30^, genotoxicity^27^	^8^ Beukes et al., 2017 ^30^ Jestoi, 2008 ^27^ Mallebrera et al., 2018
Enniatins	*F. merismoides*^8^, *F. acuminatum*^30^, *F. arthrosporioides*^30^, *F. avenaceum*^30^, *F. compactum*^30^, *F. culmorum*^30^, *F. equiseti*^30^, *F. kyushuense*^30^, *F. langsethiae*^30^, *F. lateritium*^30^, *F. oxysporum*^30^, *F. poae*^30^, *F. sambucinum*^30^, *F. scirpi*^30^, *F. sporotrichioides*^30^, *F. torulosum*^30^, *F. tricinctum*^30^, *F. venenotum*^30^	Non- ribosomal peptide	Antimicrobial activity^30^, insecticidal activity^30^, cytotoxicity^30^, phytotoxicity^30^	^8^ Beukes et al., 2017 ^30^ Jestoi, 2008
Fusaric acid	*F. proliferatum*^1^, *F. verticillioides*^1^, *F. fujikuroi*^1^, *F. solani*^1^, *F. temperatum*^1^, *F. subglutinans*^1, 8^, *F. musae*^1^, *F. tricinctum*^1^, *F. oxysporum*^1^, *F. equiseti*^1^, *F. sacchari*^1^, *F. concentricum*^1^, *F. andiyazi*^1^, *F. thapsinum*^8^, *F. moniliforme*^20^	Polyketide	Neurotoxicity^20^, antibacterial activity^21^, phytotoxicity^19^	^1^ Shi et al., 2016 ^8^ Beukes et al., 2017 ^19^ Stipanovic et al., 2011 ^20^ Porter et al., 1995 ^21^ Bacon et al., 2006
Fusarin C	*F. avenaceum*^9^, *F. verticillioides*^8^, *F. moniliforme*^42^, *F. graminearum*^43^, *F. culmorum*^43^, *F. crookwellense*^43^, *F. sporotrichioides*^43^*, F. poae*^43^*, F. tricinctum*^43^*, F. avenaceum*^43^	Polyketide	Estrogenic agonist^40^, carcinogenicity^40^	^8^ Beukes et al., 2017 ^9^ Bottalico and Perrone, 2002 ^42^ Gelderblom et al., 1984 ^43^ Thrane, 1988 ^40^ Sondergaard et al., 2011
Equisetin	*F. equiseti*^2^, *F. pallidoroseum*^2^, *F. heterosporum*	Polyketide	Antibacterial activity^46^, phytotoxicity^2^, antiviral activity^24^ cytotoxicity^24^, fungicidal activity^24^	^2^ Wheeler et al., 1999 ^24^ Burke et al., 2005 ^46^ Vesonder et al., 1979
Fumonisins	*F. proliferatum*^1, 8^, *F. verticillioides*^1, 8^, *F. fujikuroi*^1, 8^, *F. solani*^1^, *F. andiyazi*^8^, *F. anthophilum*^8^, *F. globosum*^8^, *F. napiforme*^8^, *F. nygamai*^8^, *F. oxysporum*^8^, *F. pseudonygamai*^8^, *F. subglutinans*^8^, *F. thapsinum*^8^, *F. temperatum*^8^	Polyketide	Carcinogenicity^23^, neurotoxicity^23^, hepatotoxicity^23^	^1^ Shi et al., 2016 ^8^ Beukes et al., 2017 ^23^ Schertz et al., 2018
Fusaproliferin	*F. globosum*^30^, *F. guttiforme*^30^, *F. konzum*^30^, *F. proliferatum*^30^, *F. pseudocircinatum*^30^, *F. pseudonygamai*^30^, *F. subglutinans*^30^, *F. verticillioides*^30^	Sesquiterpene	Phytotoxicity^30^, insecticidal activity^30^, cytotoxicity^30^, teratogenicity^30^	^30^ Jestoi, 2008
Culmorin	*F. culmorum*^44^, *F. graminearum*^44^, *F. crookwellense*^44^, *F. venenatum*^44^, *Fusarium praegraminearum*^44^	Sesquiterpene	antifungal and phytotoxic properties^44^, weak cytotoxicity^44^, weak teratogenicity^44^	^44^ Weber et al., 2018
Zearalenone	*F. culmorum*^1, 3, 4, 9^, *F. meridionale*^1^, *F. graminearum*^1, 3^, *F. equiseti*^3, 4^, *F. crookwellense*^9^, *F. oxysporum*^8^, *F. pseudograminearum*^8^, *F. semitectum*^8^	β-resorcyclic acid lactone	Non-steroidal estrogen^14^, immunotoxicity^14^, hepatocarcinogenicity^45^, nephropathy^45^, hematotoxicity^45^	^1^ Shi et al., 2016 ^3^ Thrane, 1986 ^4^ Hestbjerg et al., 2002 ^8^ Beukes et al., 2017 ^9^ Bottalico and Perrone, 2002 ^14^ Kuiper-Goodman et al., 1987 ^45^ Buranatragool et al., 2015
Butenolide	*F. culmorum*^3^, *F. sporotrichoides*^3^, *F. tricinctum*^3^, *F. graminearum*^10^	Lactones	Cytotoxicity^11^	^3^ Thrane, 1986 ^10^ Harris et al., 2007 ^11^ Wang et al., 2006
Moniliformin	*F. avenaceum*^3, 9^, *F. acuminatum*^8^, *F. anthophilum*^8^, *F. chlamydosporum*^8^, *F. culmorum*^8^, *F. fujikuroi*^8^, *F. napiforme*^8^, *F. nygamai*^8^, *F. oxysporum*^8^, *F. proliferatum*^8^, *F. pseudonygamai*^8^, *F. semitectum*^8^, *F. subglutinans*^8^, *F. thapsinum*^8^, *F. temperatum*^8^, *F. verticillioides*^8^, *F. acutatum*^30^, *F. arthrosporioides*^30^, *F. begoniae*^30^, *F. beomiforme*^30^, *F. bulbicola*^30^, *F. concolor*^30^, *F. denticulatum*^30^, *F. dlaminii*^30^, *F. equiseti*^30^, *F. fusarioides*^30^, *F. lactis*^30^, *F. nisikadoi*^30^, *F. phyllophilum*^30^, *F. pseudoanthophilum*^30^, *F. pseudocircinatum*^30^, *F. ramigenum*^30^, *F. redolens*^30^, *F. retuculatum*^30^, *F. sacchari*^30^, *F. sambucinum*^30^, *F. sporotrichioides*^30^, *F. tricinctum*^30^	Cyclobutane	Phytotoxicity^30^, cytotoxicity^30^	^3^ Thrane, 1986 ^8^ Beukes et al., 2017 ^9^ Bottalico and Perrone, 2002 ^30^ Jestoi, 2008

## Why are Mycotoxins Made?

### Mycotoxins in Fungal-Bacterial Battles

The advances in sequencing and bioinformatic technologies have shown that fungi, including the *Fusarium* spp. possess a large number of biosynthetic gene clusters (BGCs) with the potential to produce a myriad of secondary metabolites. One of the most powerful ways to activate BGCs is by co-cultivation or mixed fermentation of two or more microbes. Microbial interactions that have led to up-regulation of known metabolites or to discovery of novel natural products are numerous (Schroeckh et al., 2009; Bao et al., 2017). Co-cultivation of *F. tricinctum* and *F. begoniae* has led to the identification of subenniatins A and B, which have been reported to be the precursors of enniatins (Wang et al., 2013). Co-cultivation of *F. tricinctum* and *Bacillus subtilis* also led to the identification of a few novel metabolites such as macrocarpon C, 2-(carboxymethylamino)benzoic acid and citreoisocoumarinol [(Ola et al., 2013), and references therein]. *F. oxysporum* MSA 35 is an antagonist to wilt-causing *F. oxysporum* species. The antagonism has been at least in-part attributed to small volatile organic compounds produced by the fungus only when it is in association with ectosymbiotic bacteria (Minerdi et al., 2011) The exposure of *Serratia plymuthica*, to volatiles produced by *F. culmorum* upregulated the production of the volatile bacterial terpene sodorifen (Schmidt et al., 2017).

Several emerging studies place mycotoxins directly in the microbial battleground. One elaborate interaction involves the wilt-causing phytobacterium *Ralstonia solanacearum* and *F. fujikuroi*, the causal agent of foolish seedling disease in rice. The bacterium produces the lipopeptide ralsolamycin, which induces developmental changes in many fungal species resulting in chlamydospore formation. These chlamydospores are subsequently colonized by *Ralstonia* (Spraker et al., 2016). In response to this invasion, the *F. fujikuroi* responds with an increase in localized production of bikaverin and beauvericin, which together show additive antibacterial activity against *Ralstonia* (Spraker et al., 2018). Fusaric acid, a mycotoxin produced by numerous *Fusarium* species, has antibacterial activity (Table 1). Fusaric acid can sequester iron which has been suggested as a mechanism of toxicity to bacteria (Ruiz et al., 2015). Production of the siderophores, pyoverdine, and ennantio-psychelin, by *Pseudomonas protegens* has been demonstrated to contribute to the resistance of the bacterium to fusaric acid (Ruiz et al., 2015). Further, pyoverdine has been shown to contribute to successful survival in soil (Drehe et al., 2018). Fusaric acid has also been reported to repress the expression of biosynthetic genes involved in the production of 2,4-diacetylphloroglucinol, an antimicrobial polyketide made by *Pseudomonas fluorescens*, both *in vitro* as well as in the wheat rhizosphere (Notz et al., 2002). *Pseudomonas protegens* exhibits antibiosis against *F. verticillioides* which has been primarily attributed to the production of pyrrolnitrin, rhizoxin, and 2,4-diacetylphloroglucinol. Fusaric acid has been shown to reduce the antibiosis by *P. protegens* against *F. verticillioides* (Quecine et al., 2016). Thus, mycotoxins form an integral part of microbial interactions where they may offer protection from competing or invading microbes.

### Mycotoxins as Communication Signals in Quorum and Biofilm Formation

Quorum sensing is an important mechanism by which bacteria and fungi regulate developmental programs including biofilm formation and expression of virulence proteins through alteration of gene expression patterns based on population densities. Several studies have demonstrated how other microbes and their metabolites can interfere in quorum sensing and biofilms. Fungal secondary metabolites, including those produced by *Fusarium* spp., are involved in disrupting quorum signaling in bacteria (Martín-Rodríguez et al., 2014) (Figure 1B). Fusaric acid acts as a quorum quencher of acyl homoserine lactone molecules at low concentrations against the biocontrol agent *Pseudomonas chlororaphis* (van Rij et al., 2005). At higher concentrations, fusaric acid inhibits the production of the antifungal metabolite phenazine-1-carboxamide by the bacterium (van Rij et al., 2005). In addition, two other mycotoxins, zearalenone and fumonisin, have been demonstrated to inhibit quorum sensing in the bacterium *Chromobacterium violaceum* (Bacon et al., 2017). Diketopiperazines derived from gram-negative bacteria have been shown to regulate quorum-dependent phenotypes (Holden et al., 2002), possibly implicating diketopiperazine-like mycotoxins (gliotoxin, roquefortines among others) as additional quorum modulating molecules (Bacon et al., 2017). Taken together with section Mycotoxins in Fungal-Bacterial Battles, these instances indicate that mycotoxins may be synthesized in response to microbial signals in the ecological landscape, while also serving as interspecies signals themselves.

Mixed bacterial-fungal biofilms have increasingly come under scrutiny, especially in clinical settings. *Candida* spp. of fungi contribute to the majority of infections related to medical implant devices, where biofilm formation is a major contributor (Wargo and Hogan, 2006). A recent study has reported a bacterial exopolysaccharide offering antifungal resistance to *Candida* in an oral biofilm (Kim et al., 2018). *Pseudomonas aeruginosa* and *Aspergillus fumigatus* have also been reported to form mixed biofilms (Zheng et al., 2015). Phenazine-derived metabolites from the bacterium have been shown to regulate the developmental shifts of the fungus in co-cultured biofilms (Zheng et al., 2015). The *Fusarium* mycotoxin zearalenone has been shown to reduce *Candida* biofilm formation (Rajasekharan et al., 2018) and the *Penicillium expansum* mycotoxin patulin has been reported to modulate biofilm formation by *P. aeruginosa* and *Achromobacter sp*. (Liaqat et al., 2010). Therefore, mycotoxins may play vital roles in communication and/or microbial assembly processes that lead to successful formation of mixed biofilms in varied niches.

### Mycotoxins in Intra-kingdom Fungal Interactions

A significant increase in the levels of deoxynivalenol (DON) and zearalenone produced by *F. culmorum* has been reported upon co-culture with the fungus *Alternaria tenuissima* (Müller et al., 2012). An endophytic strain of *F. verticilloides* has been shown to reduce the corn smut disease caused by *Ustilago maydis* (Lee et al., 2009) which has in part been correlated to the fusaric acid-mediated repression of growth of *U. maydis* (Jonkers et al., 2012). *Trichoderma* species are well-known mycoparasites of several fungi including the *Fusarium* spp. (Chérif, 1990). *Trichoderma* spp. secrete cell wall degrading enzymes like chitinases and glucanases to aid in parasitism (de la Cruz et al., 1995). DON production by *F. culmorum* and *F. graminearum* strains has been reported to repress expression of the chitinase gene (encoding the N-acetyl-β-d-glucosaminidase) in *Trichoderma atroviride* (Lutz et al., 2003). *Paraconiothyrium variabile* is a plant endophytic fungus that is antagonistic to *F. oxysporum* (Combès et al., 2012). This antagonism has been attributed to *F. oxysporum*-induced production of 13-oxo-9,11-octadecadienoic acid by the endophyte. This metabolite downregulated the production of beauvericin in *F. oxysporum* (Combès et al., 2012). In the soil environment, it has been shown that *F. oxysporum* can repress the production of aflatoxin by *Aspergillus flavus* leading to a higher accumulation of the *Fusarium* mycotoxin fumonisin (Falade et al., 2016). Thus, mycotoxins may be involved in specific interactions of fungi with each other where they may offer ecological advantages to the interacting species.

### Mycotoxins in Improving Pathogen Fitness and Pathogenicity

Although secondary metabolites are not “required” for the growth and development of fungi, they function as fitness factors. Mycotoxins have been shown to contribute to the pathogenicity, aggressiveness and/or virulence of fungi. Fusaric acid has been reported to enhance the virulence of *F. oxypsorum* in both plant and animal hosts (López-Díaz et al., 2018). Mutants of *F. avenaceum* that lacked the ability to synthesize enniatin showed decreased virulence when infected on potato tubers (Herrmann et al., 1996). On the contrary, it has also been reported that the ability of *Fusarium oxysporum f. sp. melonis* isolates to synthesize beauvericin or enniatin B does not contribute to virulence in melons (Moretti et al., 2002). DON has been shown to be produced several fold-higher in infected host tissue compared to *in vitro* cultures in *F. graminearum* and *F. pseudograminearum* (Mudge et al., 2006) and functions as an important virulence factor (Proctor et al., 1995). *F. culmorum, F. graminearum*, and *F. pseudograminearum* cause fusarium head blight as well as fusarium crown rot. However, the former two pathogens are more aggressive pathogens in head blight while *F. pseudograminearum* shows enhanced fitness as the pathogen of crown rot. This has been attributed to the differential production of DON in the different tissues (stem base vs. wheat heads; Tunali et al., 2012). In the early stages of the hemibiotrophic lifestyle of the pathogen *F. graminearum*, DON has been demonstrated to inhibit apoptosis-like programmed cell death in *Arabidospis thaliana* (Diamond et al., 2013). This suggests that mycotoxins may play a vital role in modulating host defense responses. Upon colonization and establishment of an intracellular hyphal network, DON is specifically induced during wheat spike colonization by *F. graminearum* (Voigt et al., 2007). A hypothetical model for the role of DON in establishment of infection by *F. graminearum* has been proposed (Audenaert et al., 2013). An intimate cross-kingdom interaction between *Burkholderia glumae*, a seed-borne bacterium and *F. graminearum* has been recently identified. Co-cultivation of the two microbes resulted in an increase in sporulation and DON production in *F. graminearum*, which is at least partially in response to a toxic bacterial metabolite. An overall increase in disease severity was observed upon co-infection of rice with the two pathogens. The two microbes were also found to be physically attached upon microscopic observations after co-cultivation (Jung et al., 2018). These instances highlight the strong correlation between mycotoxin production and virulence/fitness of *Fusarium* spp. It is necessary to note here that microbial interactions can also reduce the virulence of *Fusarium* species on their plant hosts, as discussed in section Microbial Interactions in Detoxification and Degradation of Mycotoxins.

## How do Microbial Interactions Modulate Mycotoxin Levels?

### Regulation of Epigenetic Modifiers During Bacterial-Fungal Interactions

Epigenetics has been steadily gaining momentum in the last few decades in the world of transcriptional regulation. There is now growing evidence that microbial communication regulates epigenetic modifiers that in turn control mycotoxin biosynthesis. The SAGA complex, conserved across eukaryotes, induces transcription of genes by mediating histone acetylation of the corresponding promoters. A study that isolated the bacterium, *Pseudomonas piscium*, from the wheat head microbiome has shown that the bacterium secretes an antifungal agent, phenazine, against *F. graminearum*. Phenazine, upon entering the fungal cell, inhibits the histone acetyl transferase module of the SAGA complex which subsequently leads to an inhibition of fungal growth and pathogenicity in addition to a complete suppression of DON biosynthesis (Chen et al., 2018). Another similar instance has been reported in *Aspergillus nidulans*—*Streptomyces rapamycinicus* association where the bacterium induces histone modification mediated by the SAGA complex which results in production of orsellinic acid and its derivatives by the fungus (Nutzmann et al., 2011). It is indeed fascinating that microbes have evolved such well-tuned, intricately regulated mechanisms of interaction.

### Microbial Interactions in Detoxification and Degradation of Mycotoxins

The literature supports the idea that mycotoxins can be important players in shaping microbial communities and their interaction with hosts. Signaling molecules are regulated “coinage” and need to be recycled—through various chemical transformation processes—to maintain homeostasis in the community. Thus, it is not surprising that there are several examples of degradation of *Fusarium* mycotoxins mediated by microbes, plants and insects (Figure 1C).

Bacteria have been shown to contribute to reduction of *Fusarium* mycotoxin accumulation in grains. Preventative application of *Psuedomonas fluorescens* strain before inoculation with *F. culmorum* resulted in a significant reduction in Fusarium head blight as well as DON levels in infected wheat grains (Khan and Doohan, 2009). Endophytes belonging to *Paenibacillus polymyxa*, isolated from wild teosinte, have been shown to produce fusaridins which contribute to the antifungal activity against *F. graminearum*. Co-existence of these bacteria with *F. graminearum* in grains during storage at room temperature resulted in a significant decrease in DON accumulation (Mousa et al., 2015). A recent review summarizes the different bacteria and fungi that can degrade mycotoxins including zearalenone and DON (Vanhoutte et al., 2016).

Understanding the mechanisms underlying chemical transformation of mycotoxins could pave the way toward evolving novel techniques for mycotoxin decontamination in food and feed. Several mechanisms of detoxification of DON have been studied as summarized in Figure 1C. *Aspergillus tubingenesis* NJA-1, a soil isolate, has been shown to convert DON into a less-toxic product that has been speculated to be the result of hydrolysis, based on differences in the mass of the metabolites (He et al., 2008). *Agrobacterium*–*Rhizobium* strain E3-39 converts DON into 3-keto DON (Shima et al., 1997); *Nocardioides* WSN05-2 forms the non-toxic epimer, 3-epi-DON (Ikunaga et al., 2011), *Deviosa insulae* forms 3-keto-DON (Wang et al., 2019) and *Devosia mutans* 17-2-E-8 forms both 3-keto-DON and 3-epi-DON (He et al., 2015). A recent work has provided evidence that the formation of the epimer from zearalenone proceeds with 3-keto-DON as an intermediate (Hassan et al., 2017). Rumen-associated bacteria can inactivate DON by de-epoxidation since the epoxy group is vital for the toxicity of DON. *Eggerthella* spp., isolated from chicken intestine, has been reported to de-epoxify DON over a wide range of temperatures and pH (Gao et al., 2018). The gene *Tri101*, encoding 3-O-acetyltransferase for 3-O-acetylation of the trichothecene ring has been characterized in *F. graminearum* and has been further identified in other *Fusarium* species (Kimura et al., 1998; Khatibi et al., 2011). DON-glucosides have been reported to be formed as a result of plant metabolism (Sewald et al., 1992) as well as insect metabolism (De Zutter et al., 2016). In *Arabidopsis*, it has been reported that the UDP-glycosyltransferase catalyzes transfer of glucose from UDP-glucose to the hydroxyl group at the 3-C position in DON (Poppenberger et al., 2003). Whether acetylation and glycosylation can be considered detoxification is subject to debate since these forms can be hydrolyzed to regenerate the toxins in the animal gut (Ji et al., 2016).

Zearalenone mimics estrogen upon ingestion in animals and humans resulting in sexual and reproductive abnormalities. Microbes possess the ability to degrade and inactivate zearalenone, as reviewed in Ji et al. (2016). *Clonostachys rosea* has been reported to produce a zearalenone-specific lactonase that catalyzes the hydrolysis of the lactone ring, which is followed by spontaneous decarboxylation (Utermark and Karlovsky, 2007). This has been demonstrated o be responsible for the resistance of *C. rosea* to zearalenone. *Trichosporon mycotoxinivorans* has been shown to convert zearalenone into ZOM-1 which is characterized by the opening of the ring structure at the ketone group positioned at C6' (Vekiru et al., 2010). Further, ZOM-1 has been shown to have lost the estrogenic activity (Vekiru et al., 2010). *Rhizopus arrhizus* catalyzes sulfation of the hydroxyl group at the C4 position resulting in the formation of zearalenone-4-O-sulfate conjugate (el-Sharkaway et al., 1991).

Species of *Pseudomonas* (Altalhi, 2007), *Bacillus* (Cho et al., 2010; Yi et al., 2011; Hsu et al., 2018), *Rhodoccous*, and *Streptomyces* (De Mets et al., 2018) have been reported to degrade zearalenone. Degradation may not always result in detoxification. *Acinetobacter* has been shown to secrete extracellular enzymes that oxidize zearalenone into smaller estrogenic products (Yu et al., 2011). Interestingly, a mixed culture of bacteria enriched from a coal gasification site completely degraded zearalenone but lost the capability upon purification (Megharaj et al., 1997). Although reports of degradation have emerged, the degradation products as well as biochemical and genetic mechanisms underlying these processes remain unclear. El-Nezami et al. have shown that no degradation products were observed upon culturing *Lactobacillus* strains with zearalenone although the bacteria removed the mycotoxin from the cultures. The authors were able to recover zearalenone from the bacterial cultures and suggest that the bacteria bind zearalenone in a density-dependent manner (El-Nezami et al., 2002). *Lysinibacillus* sp. isolated from chicken intestine can remove zearalenone from cultures and the process has been shown to be significantly reduced upon heat treatment. The authors suggest a potential enzymatic process that may be involved in the interaction between the bacterium and zearalenone (Wang et al., 2018). *Pseudomonas putida* ZEA-1 utilizes zearalenone as a carbon source (Altalhi, 2007).

*Burkholderia ambifaria*, a novel bacterium isolated from barley rhizosphere has been reported to be able to utilize fusaric acid as a sole carbon source (Simonetti et al., 2018). Other examples of detoxification include conversion of fusaric acid to—fusarinol by *Aspergillus tubingensis* (Crutcher et al., 2014), 4-butyl-2-carboxy-pyrimidine by *Colletotrichum* sp (Fakhouri et al., 2003), and hydroxyfusaric acid by *Mucor rouxii* (Crutcher et al., 2017).

A significant understanding of the biochemical pathways involved in detoxification processes (Carere et al., 2018) along with the biotechnological advancements may pave the path toward novel detoxification methodologies that are feasible and economical.

## Concluding Statement

The mycotoxigenic fungal species live in complex and nutrient-deficient environments—be it in soil, plant or animal hosts. The soil micro-environment often fluctuates with variations in water availability, air, light, and temperature, among other abiotic factors. Now add to this, a complex cocktail of microbes and hosts that are integral to the environment where survival of microbes heavily depends on active community participation. Mycotoxins play a significant role in the defensive strategies of mycotoxigenic fungi against the resident microbes. Interactions between microbes in such environments may involve competition or compromise where mycotoxins may serve as essential chemical language mediating communication. The host environments are usually unfriendly, thus requiring special adaptations in order for the fungi to thrive in such conditions. Several studies support a view that mycotoxins may act as signaling molecules that modulate host responses and promote successful colonization. Reports of microbes that can metabolize and detoxify mycotoxins are aplenty, highlighting the importance of examining microbial interactions to uncover strategies for mycotoxin detoxification.

In this review chapter, we have summarized existing literature that accentuate the ecological significance of mycotoxins with focus on *Fusarium* spp. The evolving knowledge on molecular and genetic mechanisms that govern mycotoxin production provides us with valuable tools to study the ecological roles of mycotoxins. This is not only an achievable goal but also has the potential to be highly rewarding. Such knowledge can facilitate development of novel strategies to control infections of mycotoxigenic fungi as well as mycotoxin contamination in food and feed.

## Author Contributions

NV along with NK conceptualized and drafted the theme of the review. NV wrote the article and NK reviewed, edited, and refined the manuscript along with NV.

### Conflict of Interest Statement

The authors declare that the research was conducted in the absence of any commercial or financial relationships that could be construed as a potential conflict of interest.
